# Impact of chronic obstructive pulmonary disease on survival and neurologic outcomes in adults with in-hospital cardiac arrest

**DOI:** 10.1371/journal.pone.0259698

**Published:** 2021-11-29

**Authors:** Asem Qadeer, Puja B. Parikh, Charles A. Ramkishun, Justin Tai, Jignesh K. Patel

**Affiliations:** Department of Medicine, State University of New York at Stony Brook, Stony Brook, NY, United States of America; Fondazione IRCCS Policlinico San Matteo, ITALY

## Abstract

**Background:**

Little data exists regarding the association of chronic obstructive pulmonary disease (COPD) on outcomes in the setting of in-hospital cardiac arrest (IHCA). We sought to assess the impact of COPD on mortality and neurologic outcomes in adults with IHCA.

**Methods:**

The study population included 593 consecutive hospitalized patients with IHCA undergoing ACLS-guided resuscitation at an academic tertiary medical center from 2012–2018. The primary and secondary outcomes of interest were survival to discharge and favorable neurological outcome (defined as a Glasgow Outcome Score of 4–5) respectively.

**Results:**

Of the 593 patients studied, 162 (27.3%) had COPD while 431 (72.7%) did not. Patients with COPD were older, more often female, and had higher Charlson Comorbidity score. Location of cardiac arrest, initial rhythm, duration of cardiopulmonary resuscitation, and rates of defibrillation and return of spontaneous circulation were similar in both groups. Patients with COPD had significantly lower rates of survival to discharge (10.5% vs 21.6%, p = 0.002) and favorable neurologic outcomes (7.4% vs 15.9%, p = 0.007). In multivariable analyses, COPD was independently associated with lower rates of survival to discharge [odds ratio (OR) 0.54, 95% confidence interval (CI) 0.30–0.98, p = 0.041].

**Conclusions:**

In this contemporary prospective registry of adults with IHCA, COPD was independently associated with significantly lower rates of survival to discharge.

## Introduction

Approximately 1% of adults hospitalized in the U.S. suffer in-hospital cardiac arrest (IHCA) [1–5]. In spite of improvements in resuscitative and post-reuscitative care, rates of survival in IHCA remain as low as 25% [1–6]. Subsequent anoxic brain injury results in even lower rates of favorable neurologic recovery in cardiac arrest survivors [1–3]. Chronic obstructive pulmonary disease (COPD) has been associated with poor clinical outcomes in multiple cardiac conditions, including ST-elevation myocardial infarction [7], atrial fibrillation [8], and peripheral arterial disease [9], and in patients undergoing cardiac procedures, including coronary artery bypass graft surgery [10], aortic arch replacement [11], and transcatheter aortic valve implantation [12]. There is a paucity of data examining the association between COPD and outcomes in the setting of out-of-hospital cardiac arrest (OHCA) [13–16]. To our knowledge, there is no data investigating the impact of COPD on outcomes in the setting of IHCA. Accordingly, we sought to study the impact of COPD on in-hospital mortality and favorable neurologic outcomes in adults with IHCA at an academic tertiary care medical center.

## Methods

We conducted a prospective observational study at an academic tertiary care medical center. All adults (age ≥ 18 years) with IHCA requiring ACLS-guided cardiopulmonary resuscitation (CPR) from 2012–2018 were included in this study. Cardiac arrest was defined as absent heartbeat and respirations requiring CPR. Patients admitted with OHCA were excluded. All in-hospital cardiac arrests at our institution are managed by an organized Rapid Response and Cardiac Arrest Team for administration of ACLS-guided CPR. Airway management is handled by a Respiratory Therapist if the patient is already intubated with manual bag technique at a rate of every 6 seconds. If not intubated, the patient is intubated by a qualified physician (in the Department of Anesthesia or Division of Pulmonary Critical Care) and airway is confirmed using color calorimeter, end-tidal CO2 monitoring and auscultation.

Demographic and medical history recorded were all obtained from patient’s electronic medical record and included age, sex, baseline medical comorbidities [i.e. coronary artery disease (CAD), prior percutaneous coronary intervention (PCI), hypertension, hyperlipidemia], psychiatric comorbidities (i.e. depression, anxiety disorder, bipolar disorder, and schizophrenia) and Charlson co-morbidity score with its respective components [i.e. prior myocardial infarction (MI), congestive heart failure (CHF), peripheral artery disease (PAD), cerebrovascular disease, dementia, COPD, connective tissue disease, peptic ulcer disease, advanced liver disease, diabetes mellitus with and without end-organ damage (e.g. nephropathy, neuropathy, retinopathy), hemiplegia, advanced chronic kidney disease (CKD), solid tumor (i.e. localized, metastatic), leukemia, lymphoma, and acquired immunodeficiency syndrome (AIDS)]. Clinical presentation data extracted included CPR duration, initial electrical rhythm, receipt of defibrillation (i.e. at any time during CPR if shockable rhythm was present) and targeted temperature management (TTM), and return of spontaneous circulation [sustained (lasting ≥ 20 minutes), unsustained (lasting < 20 minutes), or never achieved].

The primary and secondary outcomes of interest were survival to discharge and favorable neurological outcome (defined as a Glasgow Outcome Score of 4–5) at the time of discharge respectively [17]. This study was approved by the Institutional Review Board of Stony Brook University Medical Center and a waiver of consent was obtained to utilize patient data.

Chi-squared test (or Fisher’s exact test if applicable) and student’s t test were used to compare categorical and continuous variables respectively. Multivariable logistic regression analysis was performed to identify independent predictors of survival to discharge as well as favorable neurologic outcome. Predictors for the multivariable analysis were selected based upon statistical significance in the univariate analysis (p<0.1). SPSS version 23.0 (SPSS, Inc. Chicago, IL) was utilized for data analysis and a two-tailed p-value of 0.05 was regarded as statistically significant.

## Results

Of the 593 patients studied, 162 (27.3%) had COPD while 431 (72.7%) did not. **Table 1** depicts the baseline demographics and medical history in adults with and without COPD. Patients with COPD were older and more often female. They had higher Charlson Co-morbidity score with higher rates of coronary artery disease, hypertension, congestive heart failure, and cerebrovascular disease. **Table 2** describes clinical presentation and management of IHCA in the presence and absence of COPD. Initial rhythm, duration of CPR, and rates of defibrillation, ROSC, and receipt of TTM were similar in both groups.

**Table 1:** Demographics and baseline medical history.

	No COPD	COPD	p
	(n = 431)	(n = 162)	value
Age (years)*	66.3 ± 16.3	69.4 ± 13.9	0.033
Male gender*	279 (64.7%)	85 (52.5%)	0.006
Coronary Artery Disease*	170 (41.8%)	85 (57.8%)	0.001
**Prior Myocardial Infarction**	60 (15.0%)	31 (21.5%)	0.072
**Prior Percutaneous Coronary Intervention**	110 (26.8%)	49 (32.9%)	0.160
Hypertension*	268 (63.2%)	116 (75.8%)	0.005
Hyperlipidemia*	189 (44.9%)	89 (58.9%)	0.003
**Diabetes Mellitus**			
** Without End Organ Damage**	87 (20.2%)	34 (22.4%)	0.568
** With End Organ Damage**	68 (15.9%)	34 (22.7%)	0.061
Congestive Heart Failure*	131 (32.2%)	81 (52.3%)	<0.001
**Peripheral Artery Disease**	43 (10.6%)	22 (16.1%)	0.092
Cerebrovascular Disease*	38 (8.9%)	22 (14.7%)	0.048
**Advanced Chronic Kidney Disease**	119 (27.9%)	55 (35.9%)	0.064
**Advanced Liver Disease**	10 (2.3%)	5 (3.4%)	0.551
**Leukemia**	18 (4.2%)	8 (5.4%)	0.552
**Malignant Lymphoma**	11 (2.6%)	6 (4.0%)	0.365
**Metastatic Solid Tumor**	35 (8.2%)	7 (4.8%)	0.170
**Connective Tissue Disease**	15 (3.5%)	6 (4.0%)	0.780
Charlson Comorbidity Score*	5.0 +/-2.8	6.4 +/- 2.7	<0.001
**Dementia**	22 (5.3%)	8 (5.4%)	0.947
**Depression**	32 (7.7%)	14 (9.5%)	0.490
**Anxiety Disorder**	28 (6.7%)	13 (8.8%)	0.400
**Bipolar Disorder**	7 (1.7%)	4 (2.7%)	0.452
**Schizophrenia**	8 (1.9%)	4 (2.6%)	0.529

**Table 2:** Clinical presentation of cardiac arrest.

	No COPD	COPD	p
	(n = 431)	(n = 162)	value
**Initial rhythm**			0.126
** VF/VT**	74 (17.7%)	23 (14.9%)	
** PEA/Asystole**	328 (78.5%)	119 (77.3%)	
** Other**	16 (3.8%)	12 (7.8%)	
**Defibrillation**	168 (39.1%)	53 (32.7%)	0.058
**CPR Duration (minutes)**	23.4 ± 22.0	20.3 ± 15.2	0.093
**Return of Spontaneous Circulation**			0.800
** Never achieved**	169 (39.3%)	67 (41.6%)	
** Unsustained (< 20 minutes)**	41 (9.5%)	13 (8.1%)	
** Sustained (> 20 minutes)**	220 (51.2%)	81 (50.3%)	
**Targeted Temperature Management**	48 (18.6%)	18 (19.1%)	0.908

VF = ventricular fibrillation; VT = ventricular tachycardia; PEA = pulseless electrical asystole; CPR = cardiopulmonary resuscitation.

**Fig 1:**
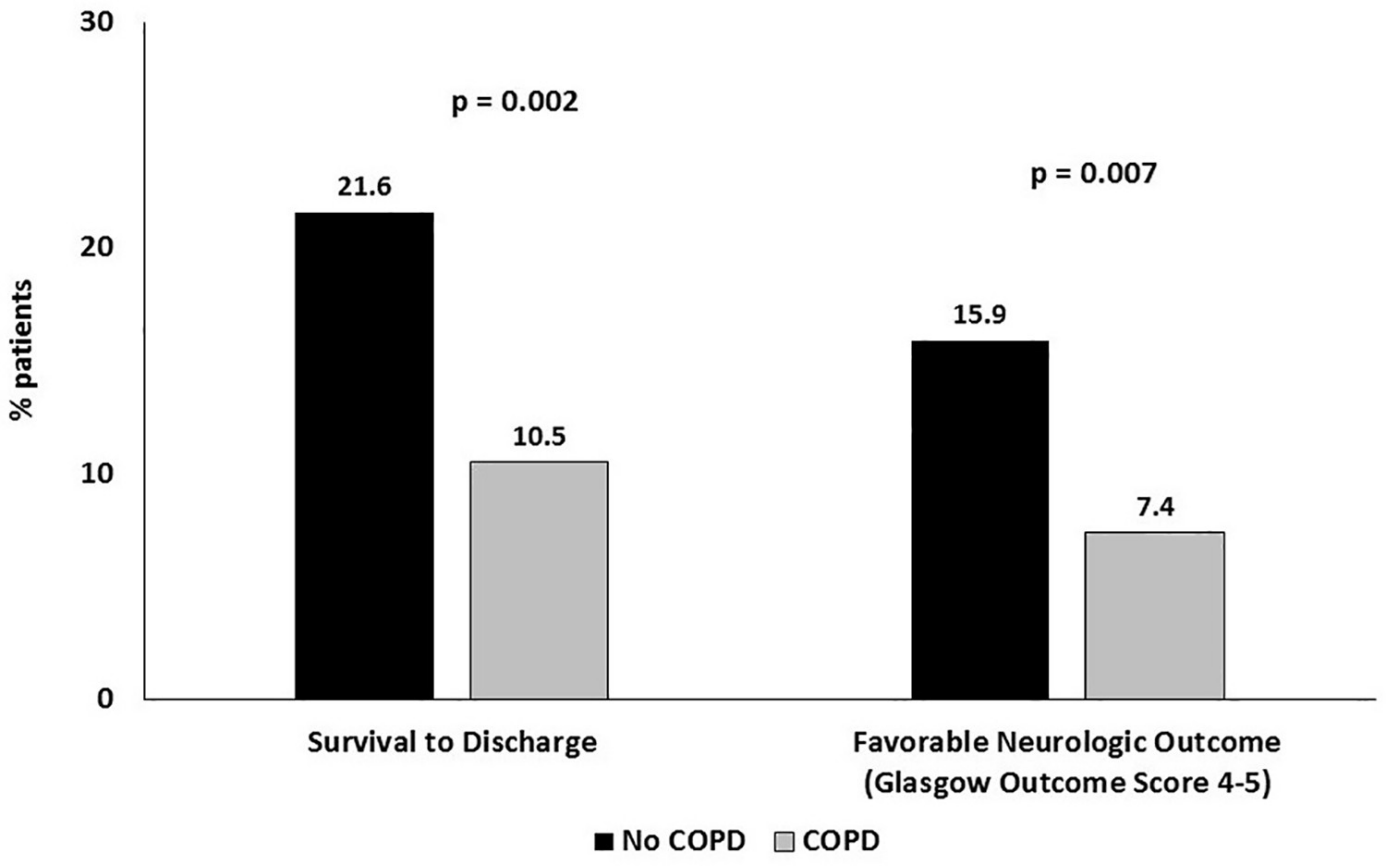
Rates of (A) In-Hospital Mortality and (B) Favorable Neurologic Outcomes in the Presence and Absence of Chronic Obstructive Pulmonary Disease (COPD).

With respect to outcomes, patients with COPD had significantly lower rates of survival to discharge (10.5% vs 21.6%, p = 0.002) and favorable neurologic outcomes (7.4% vs 15.9%, p = 0.007) compared to those without COPD (**Fig 1**). In multivariable analyses, COPD was independently associated with lower rates of survival to discharge [odds ratio (OR) 0.54, 95% confidence interval (CI) 0.30–0.98, p = 0.041] but not favorable neurologic outcome (OR 0.57, 95% CI 0.29–1.11, p = 0.096) (**Table 3**).

**Table 3:** Multivariable analysis.

	Odds	95% Confidence	p
	Ratio	Interval	value
***Survival to Discharge*** ^1^			
**Chronic obstructive pulmonary disease**	0.54	0.30–0.98	0.041
**Female gender**	0.52	0.32–0.85	0.009
***Favorable Neurologic Outcome*** ^2^			
**Chronic obstructive pulmonary disease**	0.57	0.29–1.11	0.096
**PEA/Asystole (versus VF/VT)**	0.50	0.27–0.91	0.023
**Prior myocardial infarction**	2.28	1.21–4.30	0.011
**Diabetes mellitus with end organ damage**	0.39	0.16–0.90	0.028

^1^Model included chronic obstructive pulmonary disease, age, gender, peripheral arterial disease, diabetes mellitus with end organ damage.

^2^Model included chronic obstructive pulmonary disease, age, gender, prior myocardial infarction, diabetes mellitus with end organ damage, initial rhythm.

## Discussion

To our knowledge, this is the first study to highlight the association between COPD and survival and neurologic outcomes specifically in the setting of IHCA. Several findings are noteworthy in this contemporary prospective study of adults with IHCA. First, nearly 30% of adults with IHCA have concomitant COPD. Second, initial rhythm and rates of defibrillation and ROSC are similar in the presence and absence of COPD. Finally, COPD is independently associated with nearly 2-fold lower rates of survival to discharge but no significant difference in favorable neurologic outcome.

While no other data to our knowledge exists regarding the impact of COPD on outcomes in IHCA, there are a few studies that have examined COPD’s association with outcomes in the setting of OHCA [13–15]. In one large retrospective registry of nearly 3,000 patients with OHCA secondary to ventricular tachyarrhythmias, COPD was present in less than 10% of patients [13]. COPD was associated with lower rates of VF (28% vs 39%, p = 0.001), and was independently associated with higher rates of 2-year all-cause mortality [hazard ratio (HR) 1.245; 95% CI 1.001–1.549; p = 0.001] [13]. The Danish Cardiac Arrest Registry of adults with OHCA demonstrated that over 80% of COPD patients had a non-shockable initial rhythm and that incremental severity of COPD (i.e. mild, moderate, severe) was associated with increasing prevalence of a non-shockable initial rhythm [14]. Similar to our study, patients with COPD in this Danish registry were noted to be older, less likely male, and with higher prevalence of other comorbidities. COPD patients with OHCA were less likely to have witnessed arrests and bystander CPR. While non-COPD patients experienced significant improvements in 30-day survival from 2001 to 2011 (from 3.5% to 13.0%, p<0.001), no significant change was observed in 30-day survival in COPD patients (from 3.7% to 2.1%, p = 0.27) [16].

COPD has been found to be associated with increased sudden cardiac death (SCD) risk in the community. In the Oregon Sudden Unexpected Death Study, which compared adult SCD case subjects with geographic control subjects with coronary artery disease, SCD case subjects were more likely than control subjects to have COPD (31% vs. 13%, p < 0.0001) [18]. In multivariable analysis, COPD was independently associated with over 2-fold higher rates of SCD (OR 2.2, 95% CI 1.4 to 3.5; p < 0.001) [18]. Data from the Rotterdam study, a population-based cohort study, demonstrated that COPD was associated with an increased risk of SCD (age- and sex-adjusted hazard ratio, HR, 1.34, 95% CI 1.06–1.70) [19]. The risk especially increased in persons with frequent exacerbations five years after the diagnosis of COPD [19]. Whether the heightened risk of cardiac arrest and mortality in COPD patients is related to absence of beta blocker use due to adverse effects (i.e. bronchoconstriction) is not well known.

Although smoking status was not directedly tracked in our current study, previous studies have shown that smoking has been independently associated with three-fold higher rates of survival to discharge with good neurologic outcome in adults with cardiac arrest treated with TTM compared to nonsmokers (OR 3.54, 95% CI 1.41–8.84, p = 0.007), even after adjusting for age, initial rhythm, time to ROSC, bystander CPR, and time to initiation of TH [20]. Data from the Nationwide Inpatient Sample demonstrated that in adults with IHCA, smokers were more likely to have ventricular tachycardia or ventricular fibrillation as the initial rhythm, had higher rates of survival to hospital discharge (adjusted OR 1.06, 95% CI 1.05 to 1.08, p<0.001) and lower rates of poor neurologic status (adjusted OR 0.92, 95% CI 0.89 to 0.95, p<0.001) compared with nonsmokers [21].

Our study had a number of limitations. First, diagnosis of COPD was obtained from patient’s electronic medical record and so prognostic diagnostic testing including baseline pulmonary function testing, smoking history, medication use (including beta-blockers), and arterial oxygen content [22,23] was not examined in this study. Second, while approximately half of patients in the current study were in the Intensive Care Unit (ICU) at the time of arrest, the percentage of patients on non-ICU floors who were receiving telemetry monitoring was not collected in this study, nor was the duration of time from admission to the IHCA event. Third, our study population is only limited to adults with IHCA and may not be generalized other cardiac arrest populations including OHCA. Lastly, data was limited to only in-hospital outcomes and so follow-up data, including quality of life, was not obtained.

## Conclusions

In this prospective, contemporary study of adults with IHCA, COPD was independently associated with nearly 2-fold lower rates of survival to discharge. Larger scale studies examining the association of COPD in management, processes of care, and clinical outcomes in the CA population are warranted.
